# Case study of a rare form of endometriosis

**Published:** 2013-03-25

**Authors:** M Cirstoiu, O Bodean, D Secara, O Munteanu, C Cirstoiu

**Affiliations:** * Obstetrics and Gynecology Department, Bucharest University Hospital; ** Orthopedics and Traumatology Department, Bucharest University Hospital

**Keywords:** infiltrating endometriosis, pregnancy, urinary bladder

## Abstract

Endometriosis is a common, benign, chronic, estrogen-dependent disorder. The endometrial tissue implants itself outside the uterus and can be usually found in the pelvis or, in rare cases, it can be found nearly anywhere in the body. There are no pathognomonic symptoms of this disease, therefore, in some cases the tumors are incidentally discovered during surgery. Deep infiltrative endometriosis (DIE) is a rare form of this condition, which mostly affects the uterosacral ligaments, the rectovaginal space, and the upper third of the posterior vaginal wall, the bowel, and the urinary tract.

We present the case of a 29-year-old pregnant female who was diagnosed with infiltrative endometriosis during the cesarean section at 38 weeks of gestation. The tumors involving the vesicouterine peritoneum had a tendency of infiltrating the urinary bladder, but the patient had been completely asymptomatic prior to this incidental discovery. As cited by literature, the discovery and management of urinary endometriosis, as well as that of other localizations of DIE, is not based on high-level evidence data, but rather on case-series reported by surgical teams working in different centers worldwide.

## Introduction

Endometriosis is defined as the presence of endometrial glands and stroma outside the uterus. These ectopic endometrial implants are usually located in the pelvis, but can occur almost anywhere in the body. Endometriosis is a benign, chronic, estrogen-dependent disorder. It can be associated with many distressing and debilitating symptoms, such as pelvic pain, severe dysmenorrhea, dyspareunia and infertility, or it may be asymptomatic and incidentally discovered at laparoscopy or exploratory surgery [**1**]. Three types of endometriosis have been described: peritoneal superficial endometriosis, ovarian endometriomas, and deep infiltrating endometriosis (DIE). The latter usually involves the uterosacral ligaments, the rectovaginal space, the upper third of the posterior vaginal wall, the bowel, and the urinary tract [**2**]. Urinary tract DIE may be found in up to 6% of women presenting with pelvic endometriosis and may involve either the bladder or ureters. The management of urinary endometriosis, as well as that of other localizations of DIE, is not based on high-level evidence data, but rather on case-series reported by surgical teams working in different centers around the world [**3**].

## Case report

A 29-year-old pregnant female was admitted accusing contractions. She was gravida 3, para 1, with a 38 weeks developing pregnancy. 

 From her medical history during pregnancy, result the following diagnostics: placenta praevia marginalis, antiphospholipid syndrome, autoimmune thyroiditis, vitiligo, inherited trombophilia, urinary tract infections with Proteus mirabilis and Klebsiella pneumoniae, a history of GBS colpitis (Group B Streptococcus) and a pregnancy excess weight gain of +20 kg.

 Two years prior to pregnancy, the patient underwent a cervical electro-resection with the diathermal loop (ERAD) for L-SIL (low-grade squamous intraepithelial lesion of the cervix). Her personal female history includes menarche at 12 years old, regular menstrual periods, with several episodes of menstrual pain and occasional constipation. In the past three years, the patient accused cycle-dependent pain in the upper left shoulder and in the right hypochondrium. The severe pain occurred in the 2nd day of menstrual cycle and often leads to syncopal episodes. She also had two abortions for undesired pregnancies.

 During the current pregnancy, she had made regular visits to her doctor and had made usual investigations: blood and urine tests, vaginal and abdominal ultrasound, fetal monitoring, all according to gestational stages of development. Her colpitis and urinary tract infections have been treated with antibiotics according to antibiogram results. She also received 0.4 ml of enoxaparine, one daily injection, for her inherited trombophilia and high triglycerides blood levels.

 In her 38th week of gestation, she was admitted to hospital accusing contractions and vaginal bleeding. As being formerly diagnosed with placenta praevia, antiphospholipid syndrome and more autoimmune disorders, the patient was subjected to cesarean section delivery.

**Fig. 1:**
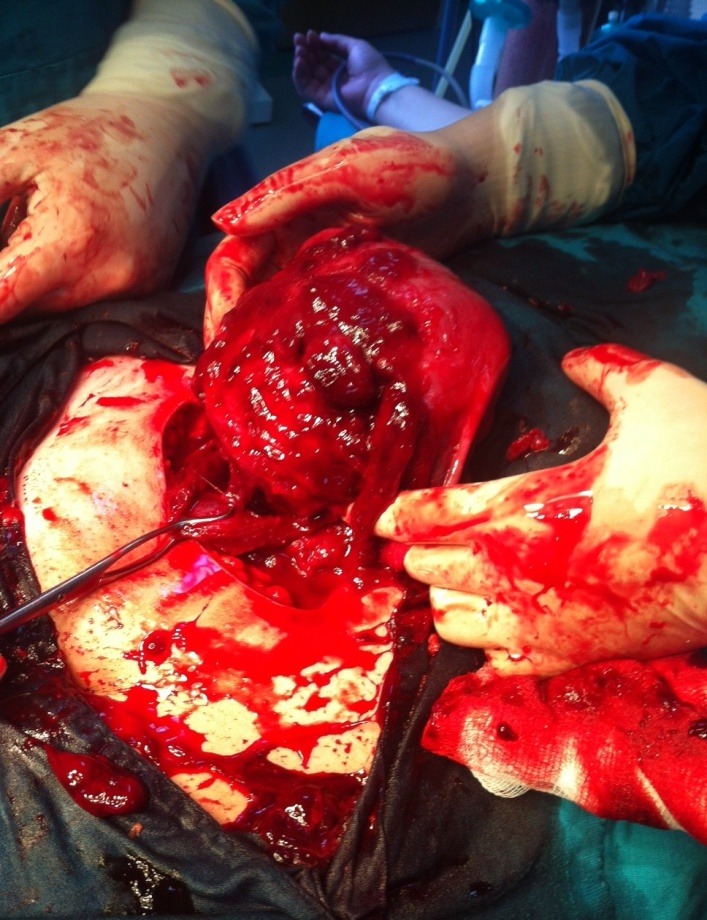
Vegetant bleeding tumors on the uterus and vesicouterine peritoneum

**Fig. 2:**
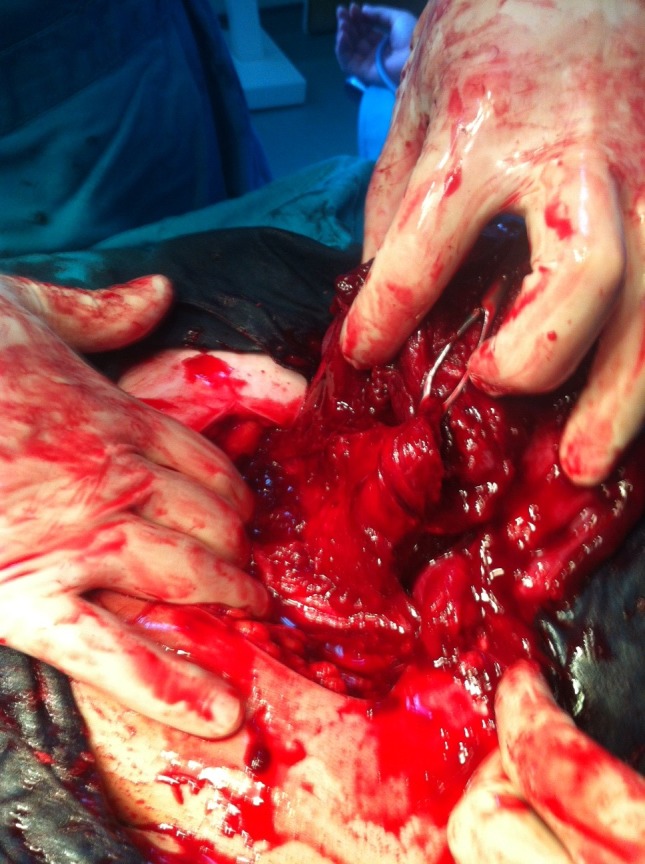
Tumor infiltrating the vesicouterine peritoneum

**Fig. 3:**
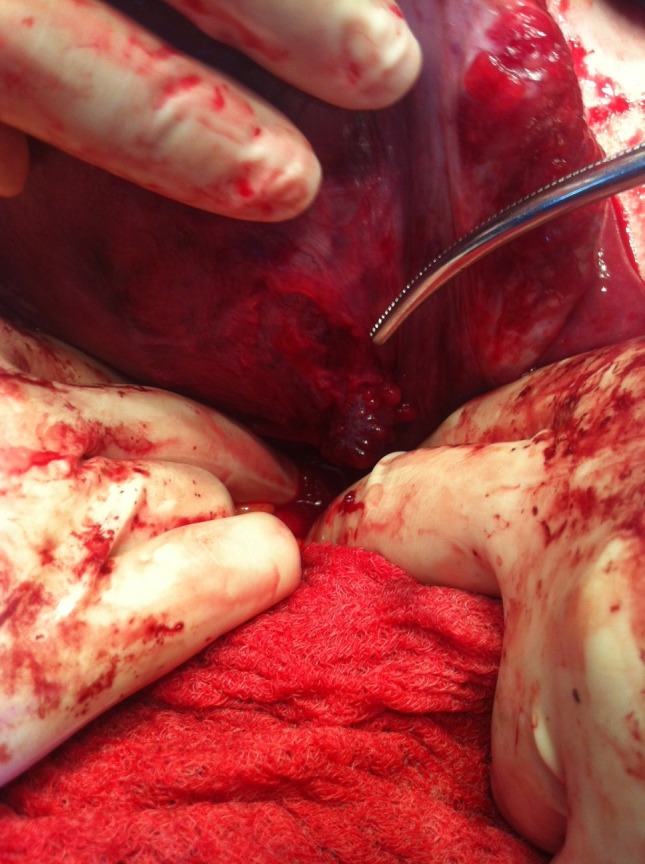
Tumor with large base of implantation, infiltrating the serous layer of the urinary bladder

 At the opening of the peritoneal cavity, we discovered 50-60 ml of blood and several bluish tumors, with vegetant and infiltrative aspect, adherent to the vesicouterine peritoneum. Some tumors were actively bleeding, having wide or narrow sites of implantation and dimensions of 5-6 cm. The operator performed viscerolysis of a part of the tumors (**Fig. 1-3**).

One tumor had a larger implantation base and appeared to infiltrate the serous coat of the urinary bladder. Excision of this tumor was performed and the coat of the bladder was sutured at the site of excision. All tumor fragments were sent to extemporaneous examination.

 Hysterotomy incision was carefully performed among the tumoral formations. The baby was extracted in cephalic presentation with no difficulty. It weighted 3250 grams and received APGAR 9. The placenta (praevia marginalis type) was extracted with difficulty as being adherent to the posterior wall of the lower uterine segment. Hemostasis was laborious in the placental bed.

 The result of the extemporaneous exam stated: “Hyperplasia of endometrial stroma with decidualized endometrium. Decidualized endometriosis".

 After the suture of the uterus was done, the team decided the excision of the rest of the tumors, which had a narrow implantation base. All tissue fragments were sent to histopathology examination along with the placenta. Hemostasis was laborious at the sites of excision and the closing of the vesicouterine peritoneum was difficult due to the tumors infiltrating into the serous coat of the urinary bladder. The further steps of the closing of the abdominal wall were normally performed, with careful but efficient hemostasis.

The evolution of the patient`s recovery in the first 48 hours after surgery was marked by a slow return of the bowel function with important abdominal meteorism. The intestinal transit was re-established with proper medication and the patient was discharged from hospital 5 days after surgery in good health conditions.

 Further, the patient had recovered well, stopped breastfeeding and was put under hormonal treatment with GnRH agonist Triptorelin (one intramuscular injection every 4 weeks for 6 months) under regular surveillance. 

## Discussion

Endometriosis is primarily found in the pelvis: on the ovaries, uterus, fallopian tubes, uterosacral ligaments, broad ligaments, round ligaments, cul-de-sac or ovarian fossa, as well as on the appendix, large bowel, ureters, bladder, or rectovaginal septum. In the case reported, we were concerned of possible endometriosis expansion into the base of the broad ligaments that can affect the normal trajectory of ureters or even intrinsic invasion cited by some authors [**11**]. Extra-pelvic locations of endometriosis are rare, but can include the upper abdomen, diaphragm, abdominal wall or abdominal scar tissue.

 Deep endometriosis is defined as a solid mass situated deeper than 5 mm under the peritoneum [**4**] and is typically characterized by multifocal locations. According to the theory of retrograde menstruation, deep endometriosis is the result of cells implanting in the most dependent areas of the pelvis, such as the spaces anterior and posterior to the uterus. These spaces serve as anatomic shelters that contain endometrial cells and prevent them from being cleared by the usual processes within the peritoneal cavity. The presence of endometrial cells elicits an inflammatory response. 

 Endometriosis tissue is biologically the same as basal endometrial tissue. Foci of endometriosis consist of glands, stroma cells, and smooth muscle; they are supplied by nerves, lymphatic vessels, and blood vessels [5,6]. Endometriosis cells express estrogen receptors (ER α/β) and progesterone receptors (PR A/B) and therefore respond to endocrine treatment [**4-7**].

 The estimated prevalence of endometriosis is 5% to 15% among all women of reproductive age. Prevalence is difficult to determine because symptoms are diverse and nonspecific and because some women are asymptomatic.

 The main manifestations are primary or secondary dysmenorrhea, bleeding disturbances, infertility, dysuria, pain on defecation (dyschezia), cycle-dependent or cycle-independent pelvic pain, nonspecific cycle-associated gastrointestinal or urogenital symptoms, constipation, diarrhea, or hematochezia, fibromyalgia and migraines. Often, no abnormalities are found and none of these symptoms is pathognomonic [**10**].

 No strong data are available concerning the prevalence of deep infiltrating intestinal endometriosis, or of endometriosis of the urinary tract. The overall prevalence of urogenital endometriosis is thought to be of 1% to 2% of the overall prevalence of endometriosis [**8-9**].

 Deep pelvic endometriosis usually involves the urinary system, with the bladder being affected in 85% of cases. Currently, the treatment is usually surgical, consisting of either transurethral resection or partial cystectomy, and eventually associated with hormonal therapy. The hormonal therapy alone counteracts only the stimulus of endometriotic tissue proliferation, with no effects on the scarring caused by this tissue. The overall recurrence rate is about 30% for combined therapies and about 35% for the hormonal treatment alone [**3**].

## Conclusions

In the present case, deep infiltrative endometriosis was incidentally found during the cesarean section in a patient with no previous clear symptoms, which adds this case to the small number of similar cases described by literature.

 The management of urinary endometriosis as well as that of other localizations of deep infiltrative endometriosis is not based on high-level evidence data, but rather on case-series reported by surgical teams working in different centers.

 Despite numerous studies, considerable controversy remains regarding the incidence, pathogenesis, natural history, and optimal treatment of this disorder.

 Deep infiltrating endometriosis and endometriosis of the urinary tract could cause long-term complications, which involve high treatment costs.
